# Prof. Davis’ Introductory Lecture at the Opening of Rush Medical College

**Published:** 1851-11

**Authors:** 


ARTICLE I.

### PROF. DAVIS' INTRODUCTORY LECTURE AT THE OPENING OF RUSH MEDICAL COLLEGE.

Prof. DAVIS' Lecture Introductory, to the regular course of Instruction in the Rush Medical College, on the 3d inst., was an able, clear and unanswerable argument in favor of increasing the facilities for students to acquire a sound and thorough Medical education, as the chief means of elevating the dignity and usefulness of the Medical Profession.

He proved, first, from reliable authority, history and reports of medical travellers, that the number of those professing and practicing the art of healing, is as great, and generally greater,in those countries where the Profession is degraded by the lowest degree of ignorance.

Secondly, that the intelligence and usefulness of the Profession in all countries, bears an intimate relation to the means afforded in those countries for acquiring a knowledge of the science and art of Medicine. That, although the relative number of practitioners to the population in this country has not increased with the rapid multiplication of Medical schools, the number of those who prepare themselves by attending upon the means of instruction before entering upon the practice, has been greatly augmented.

Thirdly, that Hospital instruction is an essential and indispensible part of the education necessary to make good and competent practitioners. To prove this position, he quoted the repeated resolves of the American Medical Association, which most emphatically insist upon it, and declare that, "College Clinics" are not recognized as substitutes for Hospital Clinical Instruction. Upon this subject, we cannot refrain from quoting the language of the author. It is so full of truthful illustration and sound reason, that we are sure it will carry conviction to every candid reader, as it did to the large and attentive audience that heard it:

"Notwithstanding these direct and oft repeated recommendations of the Association, less than one half of the whole number of Medical Colleges in this country, are so located as to afford the students in attendance on them, any opportunity for witnessing Hospital or bed-side instruction. And more than one third of the entire number of students, who annually resort to Colleges for instruction, resort to those only, where no Hospitals either do or can exist.

But, why are these things so ? Why are a greater number of students annually educated in the schools at Castleton, Woodstock and Pittsfield, than in the time-honored one at Boston, with its free access to one of the best Hospitals in the country ? Or, why do we find less than 200 annually visiting the ample Commercial Hospital of Cincinnati, while more than 300 congregate in the schools at Cleveland and Columbus in the same State ? It is not because Medical students are indifferent as to the extent and quality of the instruction which they shall receive ; neither is it because they attach no importance to demonstrative or true clinical instruction. But the true reason is found in circumstances of an entirely different character. The necessary cash expenditures for Lecture fees, boarding, &c., during a single College term in Boston or Cincinnati, for example, are very nearly twice as much as are required in either of the smaller places named. Hence, all those students who are unable to pay from $84, to $105, for Lecture fees, in addition to the increased price of board in our large cities, where alone Hospitals, worthy of the name, can be supported, must be content to forego College instruction altogether, or resort to those schools located in country villages, where lower Lecture fees and cheaper board, bring the expenditures within their means. It is thus seen that pecuniary considerations alone, deprive one third of all the Medical students in this country, of the most valuable part of Medical instruction.

In making this assertion, and the preceeding comparisons, I make no reference to the relative ability or learning of the individual professors engaged in one school or another, but I refer solely to the necessary means and facilities which their respective locations enable them to furnish the student in his search after a thorough knowledge of his profession.

The rapid multiplication of Medical schools during the last 20 years, and especially the increase of those located remote from large cities and great Hospitals, has been witnessed with regret by many of the profession ; and to the active rivalry existing among them, has been attributed the present low standard of Medical education among us. Here, however, as is too often the case, a partial comprehension of facts has caused mere effects to be mistaken for causes. If any one of the schools to which we just alluded were stricken from existence to-day, leaving the expense necessarily incurred in attending those that would be left, the same as heretofore, it would in no degree either elevate the standard of Medical education or extend the usefulness of the profession. On the contrary, it would produce directly the reverse effect on both, by placing the schools and the profession in the same relative position as they were in this country during the first quarter of the present century. Those students whose pecuniary resources would enable them to expend from $200, to $250, for each course, would continue as heretofore, to avail themselves of College instruction, while a very large proportion would be wholly unable to do so, and would consequently gain what knowledge they could from their preceptors and their text-books and enter directly on the duties of practice without ever seeing a College or a Hospital.

Instead of lessening the whole number of Medical students, as many seem to suppose, it would simply lessen the relative proportion of those resorting to Medical schools for instruction, and thereby greatly increase ignorance, in our ranks, with all its degrading consequences.

The truth is, that those Medical schools located remote from Hospitals and presenting a lower rate of Lecture fees, had their origin in, and have thus far received their support from the actual necessities of the profession; and they can be dispensed with only by obviating the necessities that gave them birth. Hence, if we would arrest the further increase of such schools as possess no adequate means for demonstrative and clinical instruction—if we would increase the relative number of those students who avail themselves of all the facilities necessary for the acquisition of a thorough Medical education—in a word, if we would increase the learning and skill of the whole profession, and thereby add greatly to its honor and usefulness, we must bring the *terms* of attendance on such schools as have access to Hospitals and every other useful appliance for communicating Medical knowledge, pecuniarily within the reach of every intelligent and upright student of Medicine. By this means, and by this alone, can the judicious recommendations of the American Medical Association, in reference to elevating the standard of requirement, extending the College term, and insisting on demonstrative and Hospital instruction, be complied with, either by Colleges or students. To clear away the obstructions that have so long barricaded the high-ways to science—to promote universal education—to elevate man intellectually and universally, is a part of the spirit of the age in which we live. And I am happy to behold around me, on my right and on my left, those who have drunk in freely and deeply of this spirit. Holding as they do, the keys of this institution, located in the great commercial metropolis of the North-West, and having unrestrained access to the only Hospital furnishing adequate means for Clinical instruction in the whole vast region north of the Ohio and west of Buffalo, they have not hesitated to open these halls to the upright and earnest inquirers after Medical knowledge, on terms *pecuniarily*, as low as the means at their command would permit. But let me presume for a moment, that they had not acted thus. Suppose they had holden fast to the ancient custom of demanding from $75 to $105 for Lecture fees, for every student who might desire admission here. What would have been the result? A favored few, to use the language of Dr. Beck, would doubtless have been found occupying these seats on the present occasion. But I speak advisedly when I say that a vast majority of those now before me would either be at their homes, where they would remain a year or two pouring over a few musty volumes, catching here and there an item of practice with their preceptors, and then launching forth as fully educated practitioners of the healing art, or they would give origin and support to half a dozen Medical Colleges located in the country, in the absence of Hospitals, where the cheapness of board, and the policy of giving *credit* for Lecture fees, would make them accessible to the great mass of students. With such a pecuniary barrier at the door, of what benefit would our Hospital be to the profession of the North-West, though it might contain a thousand patients? And of what avail would be all our means for giving instruction in General, Microscopic, and Descriptive Anatomy, Physiology and Pathology, as well as every other branch of Medical science ? And yet, for obeying the indications pointed out by the whole past history of our profession, by endeavoring to place every important and essential means for the most thorough and practical education of the student of medicine, *pecuniarily* within the reach of every earnest seeker after medical knowledge, we have been accused of lowering the *dignity* of the Profession and of *cheapening* the Medical Diploma. But do our accusers really mean that the *dignity* of our time-honored and noble profession depends on the amount of the fees which our Colleges assess as the tariff on medical knowledge, or that the *cost* of a Diploma is nothing more than the few dollars paid on the reception of the parchment? If so, precarious indeed, is all our dignity, and cheap are our boasted honors. According to this doctrine, we have only to hedge up the avenues to medical knowledge, cause the Colleges to double and treble their present rate of charging, so as to wholly exclude from their portals the great mass of young men seeking to qualify themselves for practitioners of the healing art, and we shall be the most *dignified* Profession on earth. The charge is too absurd to merit even a passing notice. When the Rush Medical College shall dispense with any of the necessary means for giving sound Medical instruction; when she shall lower, in any degree, the standard of requirements in relation to the mental attainments of those seeking her honors, let the Profession *protest* against her action ; and him who addresses you will be the first to endorse the paper. But so long as her only crime consists in rendering all the means for a thorough, demonstrative, and practical education more accessible to the profession of the North-West, I shall glory in such crime. And not only so, but I look forward with confidence to the day not far distant, when she will not only present, as she now does, all the means and appliances for the most extended and practical education, but her Lecture term shall be extended to the full period of six months, or even more. and the small pecuniary barrier now remaining, shall be still farther removed. Increase to the utmost limit every facility for acquiring Medical knowledge on the one hand, and steadfastly elevate the standard of requirements on the other, is the motto inscribed on our banner. In the matter of education, whether Medical or otherwise, I would place the active, industrious and aspiring intellect of the son of poverty on exactly the same level with the heir of fortune. The *price* of

Medical honors to both, should be drawn, not from their pockets, but from their mental toil and the ample stores of Medical knowledge which nought but their own intellectual labor could accumulate. So noble and extended a science, and so benevolent an art as Medicine never has known and never can know such a thing as a moneyed aristocracy among its votaries."

